# Clinical outcomes and risk factor analysis of early endoscopic puncture decompression for ureterocele associated with duplex kidney in children: a single-center retrospective study

**DOI:** 10.1007/s11255-023-03694-y

**Published:** 2023-07-01

**Authors:** Ye Zhang, Yin Zhang, Jiabin Jiang, Kaiping Zhang, Qihang Sun, Min Chao

**Affiliations:** Anhui Provincial Children’s Hospital, Anhui, China

**Keywords:** Ureterocele, Child, Endoscopic, Risk factors, Duplex kidney

## Abstract

**Purpose:**

The aims of this study were to analyze the clinical outcomes of treating duplex system ureteroceles with early endoscopic puncture decompression and to identify the risk factors related to outcomes to help guide future work.

**Materials and methods:**

We retrospectively reviewed the clinical records of patients with ureteroceles with duplex kidney that were treated with early endoscopic puncture decompression. Charts were reviewed for demographics, preoperative imaging, surgical indications, and follow-up data. Recurrent febrile urinary tract infections (fUTIs), de novo vesicoureteral reflux (VUR), persistent high-grade VUR, unrelieved hydroureteronephrosis, and the need for further intervention were considered unfavorable outcomes. Gender, age at surgery, BMI, antenatal diagnosis, fUTIs, bladder outlet obstruction (BOO), type of ureterocele, ipsilateral VUR diagnosed before surgery, simultaneously upper-pole moiety (UM) and lower-pole moiety (LM) obstruction, the width of ureter affiliated to UM, and maximum diameter of ureterocele were all considered potential risk factors. A binary logistic regression model was used to identify the risk factors of unfavorable outcomes.

**Results:**

A total of 36 patients with ureteroceles related to duplex kidney underwent endoscopic holmium laser puncture from 2015 to 2023 at our institution. After a median follow-up of 21.6 months, unfavorable outcomes developed in 17 patients (47.2%). Three patients underwent ipsilateral common-sheath ureter reimplantation and one patient underwent laparoscopic ipsilateral upper to lower ureteroureterostomy combined with recipient ureter reimplantation. Three patients underwent laparoscopic upper-pole nephrectomy. Fifteen patients suffered from recurrent UTIs were treated with oral antibiotics and eight of them were diagnosed de novo VUR according to voiding cystourethrography (VCUG). In univariate analysis, patients with simultaneously UM and LM obstruction (*P* = 0.003), fUTIs before surgery (*P* = 0.044), and ectopic ureterocele (*P* = 0.031) were more likely to have unfavorable outcomes. Binary logistic regression analysis showed that ectopic ureterocele (OR = 10.793, 95% CI 1.248–93.312, *P* = 0.031) and simultaneously UM and LM obstruction (OR = 8.304, 95% CI 1.311–52.589, *P* = 0.025) were identified as independent factors for unfavorable outcomes.

**Conclusions:**

Our study suggested that early endoscopic puncture decompression is not a preferred but an available treatment option to release BOO or to cure refractory UTIs. It was easier to fail if the ureterocele was ectopic or simultaneously UM and LM obstruction existed. Gender, age at surgery, BMI, antenatal diagnosis, fUTIs, bladder outlet obstruction (BOO), ipsilateral VUR diagnosed before surgery, the width of ureter affiliated to UM, and maximum diameter of ureterocele were not significantly related to the success rate of early endoscopic punctures.

## Introduction

Ureterocele is defined as cystic dilatation of the terminal ureter within the bladder, urethra, or both, and is often associated with a duplicated collecting system in children. The incidence of ureteroceles is estimated at 1 in 5000 to 1 in 500 children [1]. Due to the complexity of its pathology, there is no general agreement on a standard treatment of the duplex collecting system [2]. In recent years, transurethral incision or puncture has been gradually accepted as the first-line treatment choice [1, 3].

Several reports have described different outcomes after early decompression of ureterocele with reoperation rate of 0–100% [3, 4]. Complications include recurrent UTIs, de novo VUR, windsock phenomenon, and unrelieved hydroureteronephrosis. De novo VUR was the most common complication with an incidence of 65% [5, 6].

The watering can ureterocele puncture technique was promoted for its high decompression rate and decreased incidence of de novo VUR [7]. We have been using holmium laser energy to perform endoscopic ureterocele multiple puncture decompression since 2015, with certain curative effects. However, some of our patients still required further treatment after ureterocele puncture. The aims of this study were to analyze the clinical effects and identify the risk factors related to outcomes to help guide future work.

## Materials and methods

The clinical data of 36 patients, consisting of 9 boys and 27 girls who underwent endoscopic holmium laser ureterocele puncture with cystoscopy between 2015 and 2023 at our institution, were collected in this study. Ethical approval was obtained from our institutional ethical committee (IRB number: EYLL-2019-027). Patients with follow-up periods of less than 6 months were excluded. Included patients’ demographics, presenting symptoms, timing, and indications for surgery, collecting system duplicity, pre- and postoperative imaging features, operative recordings, and follow-up data were reviewed.

Preoperative radiographic evaluation includes urinary system ultrasonography (UUS), intravenous pyelography (IVU), VCUG, computed tomography urography (CTU), and magnetic resonance urography (MRU). The UUS findings were reviewed to determine the anteroposterior diameter of the collecting system, thickness of renal parenchyma, presence and width of ureteral dilatation, and diameter of the ureteroceles. CTU/MRU and IVU were performed to further understand the anatomical morphology of the urinary tract. VCUG findings were reviewed for the presence and type of ureterocele and the associated VUR grade. Ureteroceles were classified as orthotopic or ectopic according to imaging studies and cystoscopy findings. Nonfunctioning UM was defined, as the UM contributes to less than 10% of the overall renal function according to renogram reports. Endoscopic holmium laser puncture was performed on all patients. BOO, recurrent fUTIs, or progressive hydronephrosis were considered as the indications for operation. Patients with ureteroceles that did not cause obvious clinical symptoms or that were associated with a single collecting system, as well as ipsilateral high-grade VUR or a nonfunctioning UM, were excluded from this study. Patients with conservative treatment or whose parents strongly requested radical surgery were excluded too.

**Fig. 1:**
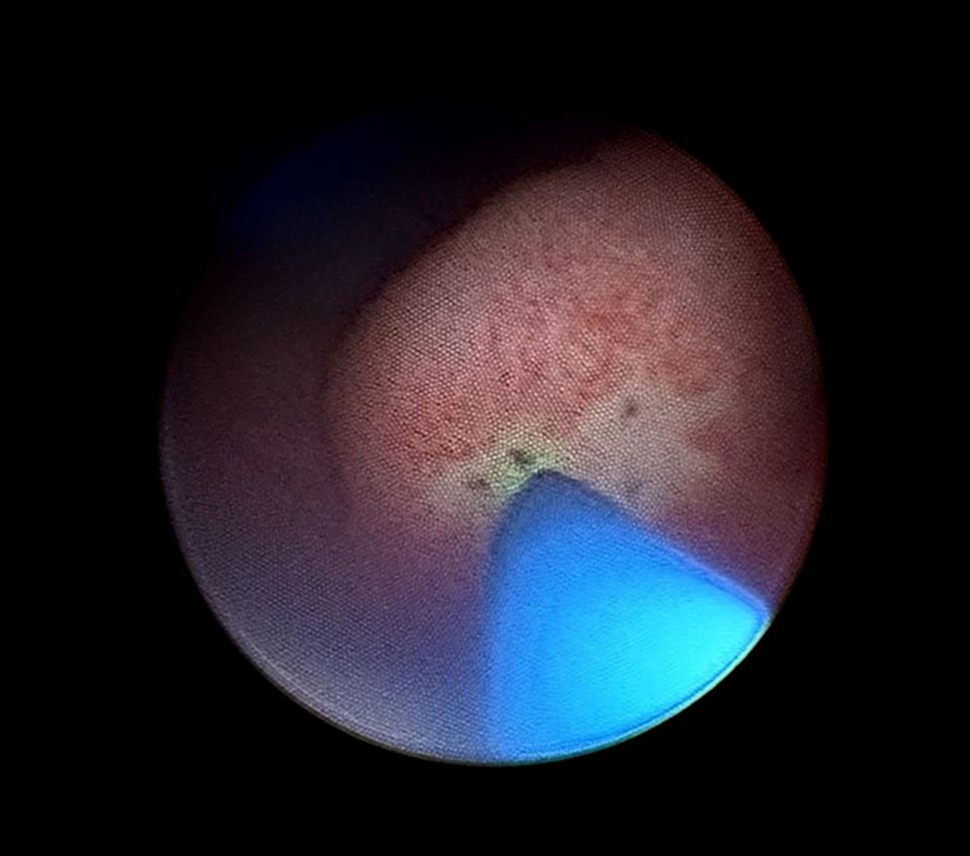
Endoscopic ureterocele multiple puncture decompression

Ureteroceles were evaluated using an 8-Fr pediatric urethrocystoscope or a 4.5 Fr ureteroscope with low pressure perfusion under general anesthesia to determine the number, position, and morphology. Then, a 200–550 µm holmium laser fiber set at 5–10 Hz and 0.8–1.0 J was used to make 10–20 puncture holes in the intravesical portion of the anterior and posterior walls of the ureterocele until stable decompression was visualized [7] (Fig. 1). A Foley indwelling catheter was placed for 24–48 h post-procedure to avoid acute bladder outlet obstruction.

Patients were discharged within 48–72 h after surgery unless refractory UTIs persisted. Antibiotic prophylaxis was prescribed pre- and postoperatively according to urinalysis and urine culture results. All patients were followed up for more than 6 months postoperatively. Subsequent UUS was routinely performed monthly for the first 3 months and as appropriate thereafter. VCUG was performed according to the patient’s clinical features, such as recurrent fUTIs. Successful decompression was defined as complete and permanent collapse or a significant reduction in the size of the ureterocele and relieved dilatation of the associated collecting system. Recurrent UTIs, de novo VUR, elevated grade of VUR, unrelieved hydroureteronephrosis, and the need for further intervention were considered unfavorable outcomes.

### Statistical analysis

All statistical analysis was performed using the Statistical Package for the Social Sciences (SPSS, Version 25.0. Armonk, NY: IBM Corp.). Data were expressed as the mean ± the standard deviation of continuous variables in normal distribution. While in skew distribution, the values were expressed as interquartile range. Student’s *t* test or the Mann–Whitney *U* test was used for continuous variables, depending on the type of distribution. Differences in distributions for categorical variables were tested with ratio and constituent ratio, Fisher’s exact test were used for comparisons between groups. *P* values of < 0.05 were considered statistically significant.

Parameters with *P* < 0.05 in univariate analyses were entered into the logistic regression analysis. A binary logistic regression analysis was performed to identify the risk factors for unfavorable outcomes. The comparisons were considered statistically significant at* P* < 0.05.

## Results

Between 2015 and 2023, the clinical data of 36 consecutive patients, constituting 9 boys and 27 girls with a median age of 3 months (range 0.36–156 months) who underwent endoscopic holmium laser ureterocele puncture at our institution, were collected in the study. All operations were performed by experienced pediatric urologists. The typical presenting clinical manifestation was fUTIs, which occurred in 53% (19/36) of the patients. Four patients complained of dysuria before surgery. Ipsilateral VUR was detected by VCUG in seven (19.4%) patients. Isolated UM obstruction was present in 17 (47%) and both UM and LM obstruction was present in 19 (53%) patients. According to UUS, the mean diameter of ureteroceles was 2.68 ± 0.71 cm,, and the mean width of the dilated ureter was 1.14 ± 0.43 cm. Ten (27.8%) patients were diagnosed with orthotopic ureteroceles and 26 (72.2%) with ectopic ones.

The mean duration of general anesthesia was 26.3 min (range 15–42 min) and the duration of hospitalization was 7.3 days (range 5–13 days).

After a median follow-up of 21.6 months (range 6–58 months), 17 patients (47.2%) had one or more complications after endoscopic puncture: two had progressive hydroureteronephrosis and 15 had recurrent UTIs. Postoperative bladder ultrasonography revealed successful ureterocele decompression in 24 (66.7%) patients and relieved hydroureteronephrosis in 21 (58.3%) patients. De novo VUR was detected in 8 patients by VCUG after 3–12 months of follow-up. Because of persistent and high-grade VUR, three patients underwent ipsilateral common-sheath ureter reimplantation and one patient underwent laparoscopic ipsilateral upper to lower ureteroureterostomy combined with recipient ureter reimplantation. Two patients underwent laparoscopic upper-pole partial nephrectomy for nonfunctioning UM owing to severe hydroureteronephrosis after endoscopic puncture. Another patient suffered from dysuria because of the windsock phenomenon and was cured by upper-pole partial nephrectomy and cystoscopic cystectomy. Ten patients were treated with oral antibiotics because of mild UTIs or asymptomatic VUR in the follow-up. None of our patients developed anastomotic stricture or incontinence, postoperatively.

In univariate analysis, we found no statistically significant differences regarding gender and age distribution or in the patients’ clinical features, including the incidence of prenatal diagnosis, BOO, and preoperative VUR between favorable and unfavorable outcomes. The diameter of the ureterocele and the width of the ureter were also not significantly different. Simultaneously, UM and LM obstruction (*P* = 0.003), fUTI (*P* = 0.044), and ectopic ureterocele (*P* = 0.031) had statistically significant effects on the unfavorable outcomes (Table 1). Cochran’s Mantel–Haenszel analysis showed that after adjusting for the influence of gender, fUTI was still associated with unfavorable outcomes (Table 2). A binary logistic regression equation was conducted by these factors. The results showed that ectopic ureterocele (OR = 10.793, 95% CI 1.248–93.312, *P* = 0.031) and simultaneously UM and LM obstruction (OR = 8.304, 95% CI 1.311–52.589, * P*=  0.025) were the independent factors which correlated with the risk of unfavorable outcomes after early endoscopic puncture decompression (Table 3).

**Table 1:** Univariate analysis of variables related to outcomes

Preoperative characteristics (*n* = 36)	Favorable outcomes (*n* = 19)	Unfavorable outcomes (*n* = 17)	*t*/*z*/*x*^*2*^	95% CI		*P*
				Lower	Upper	
Gender (%)						
Male	6	3	–			0.451
Female	13	14				
Age at surgery						
< 3 months	11	10				1.000
> 3 months	8	7				
BMI ($\overline{x}$ ± *s*)	16.79 ± 3.14	16.85 ± 2.44	− 0.052	− 1.97	1.87	0.959
Side of ureterocele						
Left	13	12				
Right	5	5				
Bilateral	1	0				1.000
Prenatal diagnosis						
No	6	7	–			0.730
Yes	13	10				
fUTI						
No	13	5	–			0.044*
Yes	6	12				
Upper urinary tract obstruction						
UM	14	3	–			0.003*
UM and LM	5	14				
BOO						
No	16	16	–			0.605
Yes	3	1				
Preoperative VUR						
No	16	13	–			0.684
Yes	3	4				
Ureterocele type						
Orthotopic	9	1	–			0.031*
Ectopic	10	16				
Ureterocele diameter, cm ($\overline{x}$ ± *s*)	2.74 ± 0.80	2.61 ± 0.59	0.572	− 0.35	0.62	0.571
Width of ureter, cm ($\overline{x}$ ± *s*)	1.08 ± 0.48	1.2 ± 0.37	− 0.842	− 0.41	0.17	0.406

* *P*<0.05 were considered statistically significant

*fUTI* febrile urinary tract infection, *VUR* vesicoureteral reflux, *UM* upper-pole moiety, *LM* lower-pole moiety, *BOO* bladder outlet obstruction

**Table 2:** Cochran’s Mantel–Haenszel analysis of the relationship between fUTI and outcomes in different genders

	OR	95% CI		*P*
		Lower	Upper	
Male	2.500	0.100	62.605	0.583
Female	5.867	1.075	32.002	0.041*
Total	5.200	1.253	21.572	0.022*
Breslow-day				0.644
Mantel–Haenszel	4.905	1.112	21.641	0.036*

* *P*<0.05 were considered statistically significant

**Table 3:** Binary logistic regression analysis of variables related to outcomes

Variables	*B*	S.E	Wald *x*^2^	df	*P*	OR	95% CI for OR	
							Lower	Upper
fUTI								
No^a^	1.317	0.928	2.014	1	0.156	3.732	0.605	23.007
Yes								
Upper urinary tract obstruction								
UM^a^	2.117	0.942	5.053	1	0.025*	8.304	1.311	52.589
UM and LM								
Ureterocele type								
Orthotopic^a^	2.379	1.101	4.672	1	0.031*	10.793	1.248	93.312
Ectopic								

* *P*<0.05 were considered statistically significant

*fUTI* febrile urinary tract infection, *UM* upper-pole moiety, *LM* lower-pole moiety

^a^Control group

## Discussion

Ureterocele is a complex malformation of the lower urinary tract and is related to the upper-pole ureter in a duplex collecting system in 80% of cases [1]. The ideal treatment continues to be debated. Different treatment strategies, including upper urinary tract reconstruction techniques and minimally invasive endoscopic techniques, all share the same goals: preserving related renal function, relieving obstruction, preventing UTIs, and maintaining continence [8]. However, reconstruction techniques may be associated with damage to the adjacent tissues and LM. Less invasive endoscopic approaches are generally accepted as the best options in acute settings early in life. Recent studies have reported that holmium–YAG laser ureterocele puncture appears to be safe and effective compared with electrocautery puncture [9, 10]. It is difficult to manipulate the procedure in a uniform way due to the great individual differences. Hence, we perform “watering can” approaches to obtain satisfactory decompression. As in this study, 19/36 (52.8%) patients achieved acceptable results. Seven of the remaining 17 patients underwent further surgery, and the others received conservative treatment.

Ureteroceles are reportedly more common in girls, especially ectopic ureteroceles [1]. This was similar to our study: 20 patients were diagnosed as having ectopic ureteroceles in 27 girls versus 6 in 9 boys. Nonetheless, the rate of unfavorable outcomes was not associated with sex distribution in this study. Recurrent UTIs pre- and postoperatively was the most common reason for patients with ureteroceles to seek medical advice [1]. In this study, 18 patients suffered from UTIs preoperatively, and the UTIs resolved in 8 patients after surgery. Postoperative UTIs occurred in 15 patients. Anatomical differences in the female urethra may make urinary tract infections easier. Stratified analysis showed that gender was not a confounding factor in the statistical analysis.

Different complications may occur after minimally invasive endoscopic procedures in different types of ureterocele. The most widely adopted classification of ureteroceles was proposed by the Committee on Terminology of the Urologic Section of the American Academy of Pediatrics. Depending on the location within the bladder, ureteroceles are classified as orthotopic or ectopic. According to previous reports, ectopic ureteroceles require carefully intervention, because they are more often associated with severe hydronephrosis or high-grade VUR [11]. Reoperation rates after ureterocele decompression in orthotopic and ectopic ureteroceles vary considerably [12, 13]. Merlini et al. considered that endoscopic ureterocele puncture is emergency therapy for intravesical obstructing ureteroceles and that treatment for ectopic ureteroceles requires additional concurrent surgery [14]. Byun et al. reported in a meta-analysis that ectopic ureterocele location, duplex renal system, and preoperative reflux are risk factors for a secondary operation after ureterocele incision [15]. However, Park stated that ectopic ureteroceles in duplex systems should be treated by primary transurethral incision as the initial treatment, as for orthotopic ureteroceles [16]. Basically, we agree with the former point. In the present study, 26 (72.2%) were diagnosed ectopic ureteroceles. The unsuccessful treatment rate was 61.5% (16/26) for ectopic ureteroceles, which was significantly higher than the rate for orthotopic ureteroceles of 10% (1/10). This revealed that ectopic ureterocele may be the potential risk factor for unfavorable outcomes of endoscopic puncture.

Anatomically, ureteroceles often distort and bend the related ureteral orifice and/or the contralateral ureteral orifice, leading to VUR or obstruction. It is reported that VUR is associated with ureteroceles in duplex systems in 50% of patients on the ipsilateral side and in less than 20% on the contralateral side [17]. In our cohort, preoperative VUR was detected in 8 patients, all of whom had reflux to the ipsilateral UM. According to previous reports, de novo VUR occurs in 50–75% of patients after endoscopic therapy [9]. Some pediatric urologists prefer to correct the VUR simultaneously [18]. However, in Nabavizdeh’s study, de novo VUR occurred in only 2 of 51 (3.9%) patients with ureterocele moieties [19]. Recently, Song reported that 15/27 (55.6%) renal units developed de novo VUR, and only 6 (40%) required a second operation [20]. Similarly, we found that de novo VUR occurred in eight (22.2%) patients of our cohort, and four patients required anti-reflux surgery. Moreover, the occurrence of preoperative VUR had no statistical significance as a risk factor of unfavorable outcomes. Dilated ureteroceles usually affect not only the related ureteral orifice, but also the ipsilateral orthotopic ureteral orifice, leading to simultaneously UM and LM obstruction. We found that unfavorable outcomes were more likely in patients with simultaneously UM and LM obstruction, and this was another crucial factor associated with unfavorable outcomes according to both univariate analysis and binary logistic regression analysis.

Pyelo-ureteral obstruction is a common presentation with ureteroceles, and each one of our patients had various degrees of hydroureteronephrosis. The ureterocele and the corresponding collecting system are often severely dilated in utero [21]. Prenatal diagnosis allows newborns to receive proper treatment shortly after birth, avoiding the risks of severe UTIs and hydroureteronephrosis. In some reports, prenatal diagnosis was positively associated with prognosis [22]. Unexpectedly, our results did not support this viewpoint.

Ureteroceles sometimes cause BOO by prolapsing into the urethra. This situation was often seen in case reports [23]. Four patients (3 orthotopic and 1 ectopic) who complained of dysuria were included in our study. After surgery, one of these patients developed a de novo ipsilateral upper-pole VUR, and she was cured after conservative treatment. Another patient suffering from dysuria because of the “windsock phenomenon” was cured by upper-pole partial nephrectomy and cystoscopic cystectomy.

## Conclusion

Early endoscopic ureterocele multiple punctures using a holmium laser is an available treatment option for patients with symptomatic ureteroceles related to duplex kidney to release BOO or to cure refractory UTIs. The therapeutic effect was not satisfied for all patients. It should be cautiously selected because of the high failure rate, especially when the patient is detected with ectopic ureteroceles or simultaneously UM and LM obstruction.

### Limitations

The limitations of this study are obvious. The nonrandomized and retrospective design means that the size and location of the ureteroceles and the number of punctures were not standardized. The other limitation is the small sample size, which may have influenced the statistical results. Another limitation is the short-term follow-up. Some asymptomatic VUR patients might become symptomatic in the further follow-up. The postoperative bladder function cannot be evaluated either [24]. Another limitation is the lack of comparison of pre- and postoperative renogram assessment. Therefore, a well-designed, randomized, prospective study with long-term follow-up is required to determine the optimal management of ureteroceles with a duplex system.

## Data Availability

The original data presented in the study are included in the article, further inquiries can be directed to the corresponding author or the first author.

## Abbreviations

UM: Upper-pole moiety
LM: Lower-pole moiety
VUR: Vesical–ureteral reflux
UTIs: Urinary tract infections
UUS: Urinary system ultrasonography
VCUG: Voiding cystourethrography
BOO: Bladder outlet obstruction
